# Sleep, circadian rhythms, and type 2 diabetes mellitus

**DOI:** 10.1111/cen.14607

**Published:** 2021-10-12

**Authors:** Gokul Parameswaran, David W. Ray

**Affiliations:** ^1^ Oxford Centre for Diabetes, Endocrinology and Metabolism University of Oxford Oxford UK; ^2^ NIHR Oxford Biomedical Research Centre John Radcliffe Hospital Oxford UK

**Keywords:** circadian clock, energy metabolism, liver, sleep, type 2 diabetes

## Abstract

Over the last 60 years we have seen a significant rise in metabolic disease, especially type 2 diabetes. In the same period, the emergence of electricity and artificial lighting has allowed our behavioural cycles to be independent of external patterns of sunlight. This has led to a corresponding increase in sleep deprivation, estimated to be about 1 hour per night, as well as circadian misalignment (living against the clock). Evidence from experimental animals as well as controlled human subjects have shown that sleep deprivation and circadian misalignment can both directly drive metabolic dysfunction, causing diabetes. However, the precise mechanism by which these processes contribute to insulin resistance remains poorly understood. In this article, we will review the new literature in the field and propose a model attempting to reconcile the experimental observations made. We believe our model will serve as a useful point of reference to understand how metabolic dysfunction can emerge from sleep or circadian rhythm disruptions, providing new directions for research and therapy.

## 1 INTRODUCTION

Diabetes mellitus is characterized by chronic hyperglycaemia arising from dysregulation of carbohydrate, lipid and protein metabolism.^1^ Type 2 diabetes mellitus (T2DM) is the most common form of diabetes, accounting for 90% of cases, affecting over 460 million worldwide with projections expecting this number to rise to over 700 million in just 25 years.^2^ The main cause of T2DM is skeletal muscle, liver and adipose tissue insulin resistance eventually giving rise to pancreatic β‐cell dysfunction and failure.^3^ These impairments result in a chronic hyperglycaemic state which, if left untreated, can cause serious complications including macrovascular and microvascular disease.^1^ Mirroring this secular rise in T2DM, over the last century, there has been an inverse decline in sleep duration.^4^, ^5^

Industrialisation and electrification allow us to spend more time indoors without needing to adhere to external patterns of sunlight. In our 24/7 society, the availability of everything all the time has meant more and more people view sleep as an inconvenience that must be overcome to satisfy work and social demands.^6^ However, this change in sleep‐wake behaviour has meant sleep and circadian rhythm disruption (SCRD) is more common than ever before. For the purposes of this article, SCRD refers to any problem associated with quality, timing and amount of sleep including pathological conditions as well as voluntary disruptions to sleep or circadian cycles.^7^ Disruptions to these rhythms can have serious consequences for health with evidence strongly implicating SCRD in increasing the risk of T2DM.^6^, ^7^, ^8^ This link between sleep and T2DM initially emerged from human laboratory studies which showed total or partial sleep restriction for a few days results in reduced glucose tolerance and impaired insulin sensitivity.^9^, ^10^, ^11^ Similarly, several large‐scale cross‐sectional studies and meta‐analyses have shown a U‐shaped relationship between the risk of T2DM and sleep duration with 7–8 h being considered optimal sleep.^12^, ^13^, ^14^ It is a surprising and consistent feature that both short or long sleep duration appears to increase the risk of T2DM, reducing the likelihood of a simple linear relationship between metabolic disease and hours of sleep.

Supporting these results, investigations into T2DM incidence in obstructive sleep apnea (OSA) patients, who exhibit increased sleep fragmentation and reduced duration, showed those with more severe sleep apnea had a higher risk of T2DM, even after controlling for other risk factors such as age and obesity.^15^, ^16^ That said we note that OSA is a complex disease, associated with chronic inflammation, and other factors may play important roles independent of the sleep loss. Aberrant timing of sleep and behavioural patterns, such as results from night shift work, has also been shown to increase the risk of diabetes.^17^, ^18^, ^19^ A less severe, but more prevalent SCRD disruption is social jet lag. This occurs when preferred sleep‐wake times are only permitted on rest days, typically with affected subjects waking earlier than they would like on workdays. People with social jet lag, and a difference of more than 2 h in wake time between rest days and workdays, have a higher incidence of T2DM.^19^, ^20^ With the incidence of insomnia rising, ~20% of the workforce doing shift work and ~70% of people suffering from social jet lag,^21^ SCRD may be an important risk factor in understanding the rise of T2DM. The different lines of evidence that help establish the link between T2DM and SCRD are summarized in Table 1.

**Table 1:** Summary of the experimental approaches and populations studied which implicate SCRD with onset of T2DM

Investigative approach linking T2DM with abnormal sleep patterns	Advantages to the approach	Disadvantages to the approach	Examples of studies using this approach
Lab‐based sleep deprivation in humans	1. Directly link sleep loss with symptoms of T2DM 2. Can control food intake or other variables 3. Can measure sleep duration rather than using self‐reports	1. Cannot be conducted for long periods of time 2. Small sample size with over‐representation of the Caucasian population 3. Mostly studies done in the lab which may affect normal sleep patterns of participants	Spiegel et al.^11^ Spiegel et al.^9^ Tsali et al.^10^
Lab‐based sleep deprivation in rodents	1. Directly link sleep loss with symptoms of T2DM 2. Can control food intake or other variables 3. Can be conducted chronically to assess effects of sleep loss	1. Animal models may not fully translate to humans 2. Protocols keeping rodents awake vary widely 3. Time‐consuming and expensive to do so only small number of animals used	Xu et al.^22^ Barf et al.^23^
Epidemiological data for sleep duration or quality	1. Very large sample sizes representing populations all over the world 2. Assesses sleep duration over long periods of time	1. Mostly relies on self‐reported sleep duration which may be unreliable 2. Difficult to control for confounding factors, e.g., changes in appetite	Liu et al.^12^ Shan et al.^13^ Muraki et al.^16^
Epidemiological data in night shift workers	1. Large sample size with diverse populations 2. Assesses sleep duration over long periods of time 3. Less reliant on the self‐reported sleeping times	1. Difficult to control for confounding factors, e.g., psychological factors or changes in appetite 2. Literature over‐represents certain jobs, especially nurses	Gan et al.^17^ Pan et al.^18^ Vetter et al.^19^
Epidemiological data in social jet lag sufferers	1. SJL is very common so results can be translated clinically more easily	1. Relies on self‐reported sleeping and wake times 2. Difficult to control for confounding factors as SJL affects appetite etc.	Koopman et al.^20^

Abbreviations: SCRD, sleep and circadian rhythm disruption; T2DM, type 2 diabetes mellitus.

### 1.1 Modelling SCRD impact on metabolic health

**Figure 1:**
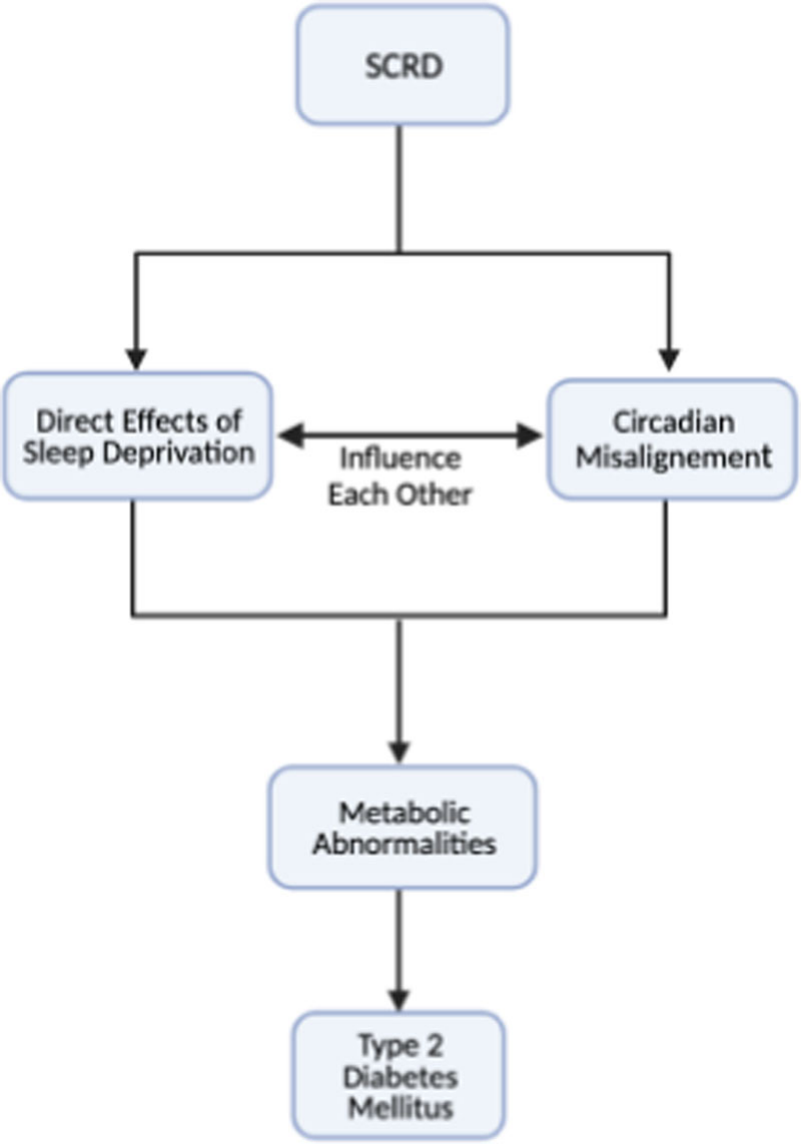
Scheme to understand how sleep and circadian rhythm disorder (SCRD) contributes to type 2 diabetes mellitus

Despite the strong evidence implicating SCRD in type 2 diabetes, the closely intertwined nature of sleep, circadian rhythms and metabolism has made it difficult to develop a clear model that explains the SCRD contribution to T2DM. In this article, we propose a simple scheme that divides the consequences of SCRD into two different arms (Figure 1). The first arm is the consequences of actual sleep deprivation, activating various processes to maintain waking with a build‐up of the homeostatic S factor (accumulates in waking, dissipates during sleep^24^). The second arm focuses on the effects of circadian misalignment arising due to changes in the timing of behavioural cycles (e.g., exercise or eating). Since both processes are closely intertwined and influence each other, disruption to either one of these axes invariably affects the other. Using our simplified scheme, however, we can begin to separate out the consequences of sleep‐circadian rhythm disruptions on metabolic health.

### 1.2 Role of sleep deprivation in T2DM

Borbély's landmark 2‐process model posits sleep regulation is controlled by the cyclical rhythm of the circadian process overlaid on the periodically rising homeostatic S factor.^24^ As per this model, the primary consequence of sleep deprivation would be further accumulation of the S factor as it cannot be fully dissipated. However, the identity of the homeostatic factor remains unclear with classical studies suggesting it is mediated by the build‐up of somnogens including adenosine, prostaglandins or interleukins.^7^ Of these, the most well‐studied is adenosine, released largely by astrocytes.^25^ Microdialysis studies have shown extracellular adenosine periodically increases in the wake‐promoting basal forebrain and declines in sleep.^26^ Furthermore, activation of adenosine receptors increases sleep while antagonists (e.g., caffeine) promote wake.^7^ Though it is clear adenosine and other somnogens contribute to sleep, they are unlikely to be the only sleep‐promoting factors. First, adenosine levels do not seem to increase significantly in many sites involved in sleep control.^27^ In addition to this, experiments using adenosine receptor KO mice have yielded inconclusive results with many animals showing only subtle or no changes in sleep.^28^, ^29^ Beyond the somnogen theory, more recent evidence suggests the S factor may be encoded by the accumulation of neuronal oxidative stress. A landmark experiment by the Miesenboeck lab investigating the dorsal‐fan‐shaped body of drosophila showed accumulation of reactive oxygen species (ROS) causes oxidation of the NADPH cofactor of the β‐subunit of the K^+^ channel Shaker to drive sleep.^30^ While this study was conducted in flies, it is likely that similar processes occur in mammals as recent theories suggest sleep's main function is providing a period of global neural quiescence to alleviate neuronal stress.^31^ Based on these theories, we can understand the consequences of S factor accumulation by considering the effects of adenosine and ROS on metabolism.

**Figure 2:**
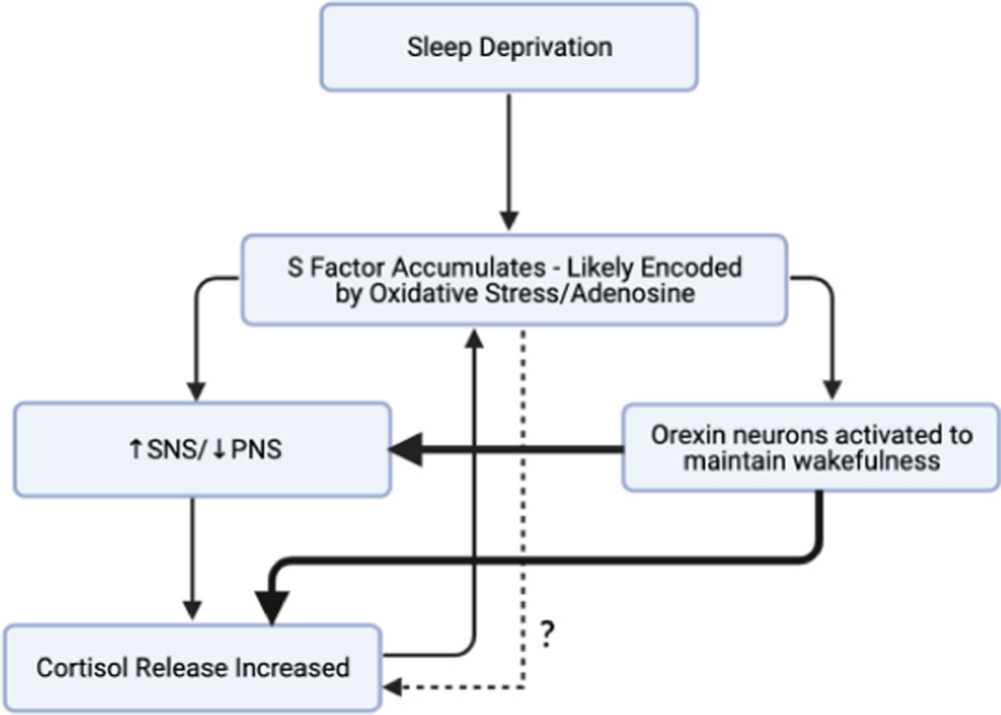
Model to understand how sleep deprivation (S factor build‐up) causes relative sympathetic nervous system dominance and increased cortisol production. PNS, parasympathetic system

Both adenosine and ROS have been shown to disrupt autonomic nervous system balance, resulting in a relative sympathetic dominance and a lower vagal tone^32^, ^33^, ^34^— widely observed effects of sleep deprivation.^11^ Although these studies focused on the cardiovascular effects, this sympathovagal imbalance has been shown to clearly affect metabolic function. The relative increase in sympathetic nervous system (SNS) activity has been shown to increase the sensitivity of adrenal glands to ACTH before sleep, elevating evening cortisol,^35^ a common observation in sleep‐deprived individuals.^11^ The increased concentrations of cortisol, at the wrong time of day, has been shown to cause further increases in neuronal stress by increasing mitochondrial reactive oxygen species generation.^36^ Aside from the effects of S factor accumulation, the neural circuits promoting wake may themselves drive cortisol release and sympathovagal imbalance. One of the best‐studied examples of this are the hypothalamic orexin neurons which increase their activity during sleep deprivation.^37^ These orexin neurons have dense projections to paraventricular (PVN) cells and autonomic nuclei, allowing them to upregulate hypothalamic‐pituitary‐adrenal (HPA)^38^, ^39^ and SNS axes while downregulating the parasympathetic system (PNS).^40^, ^41^ These findings linking sleep deprivation with cortisol release and an autonomic imbalance have been combined into a model shown in Figure 2.

**Figure 3:**
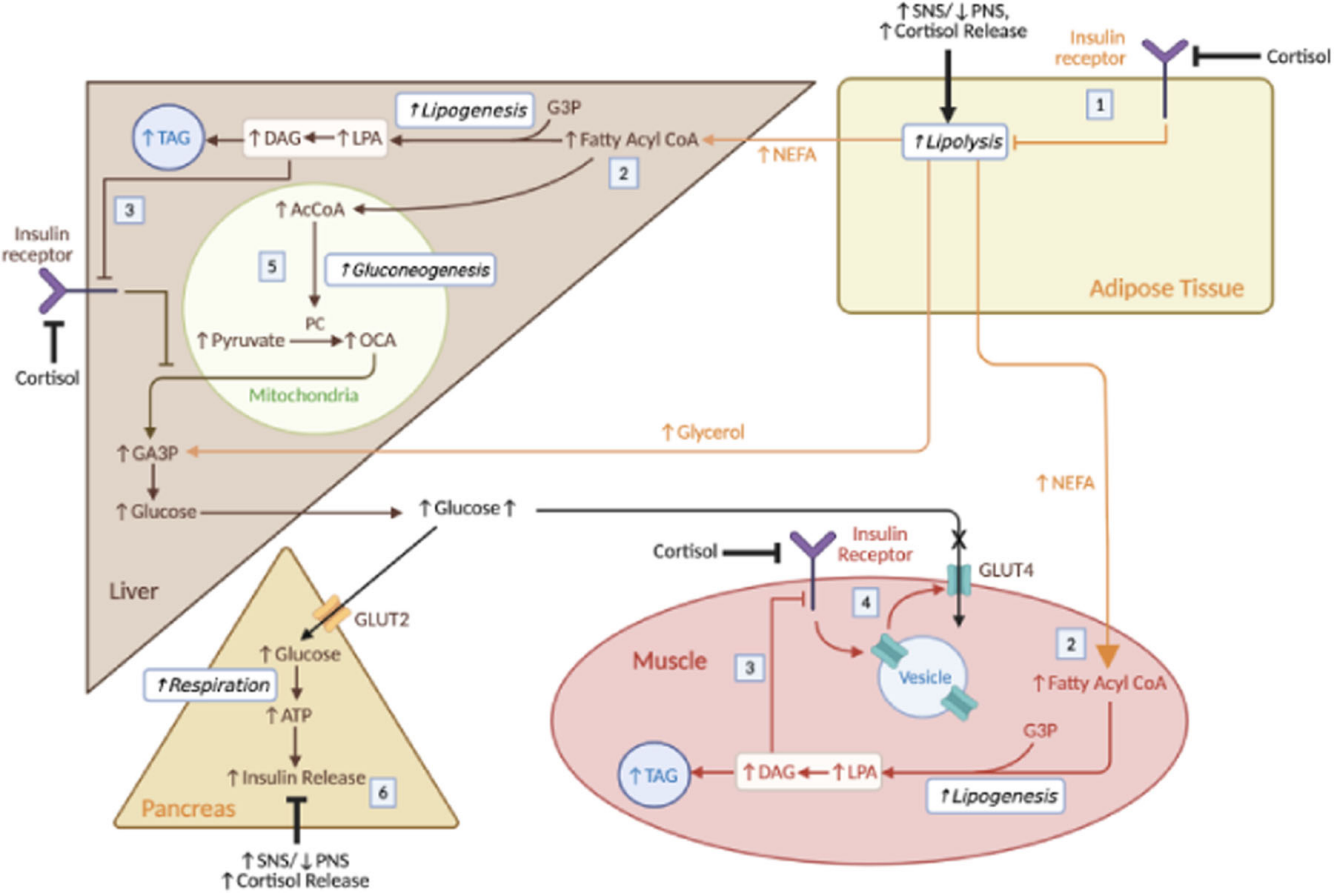
Physiological responses to sleep deprivation and impact on carbohydrate metabolism. (1) SNS dominance and cortisol drive lipolysis. The elevated cortisol levels reduce insulin sensitivity, reducing inhibition of lipolysis. (2) Lipolysis releases NEFA, which is converted into fatty acyl CoA to produce TAG in the muscle and liver. The build‐up of ectopic fat in the liver and muscle may cause other complications such as nonalcoholic fatty acid liver disease—also linked to sleep deprivation.^69^ (3) When synthesising TAG, DAG is produced which activates PKCε in hepatocytes or PKCε/PKCθ in myocytes to inhibit insulin signalling. This effect is compounded by the cortisol‐induced reduction in insulin sensitivity at these cells. (4) This reduction in insulin sensitivity in the myocytes reduces GLUT4 insertion and hence glucose uptake in muscle, increasing glucose levels. (5) In the liver, the reduced insulin sensitivity and increased levels of AcCoA drive gluconeogenesis. (6) Normally, elevated glucose drives insulin release in the pancreas, but the relative increase in SNS activity and cortisol inhibit this response. Therefore, over long periods of time, glucolipotoxicity impairs β‐cell function. Though this is not shown, the autonomic imbalance and cortisol can also directly drive inverse processes to insulin (e.g., increasing hepatic gluconeogenesis, reducing glucose uptake via GLUT4 at adipose tissue and muscle), also contributing to T2DM. PNS, parasympathetic system; SNS, sympathetic nervous system; T2DM, type 2 diabetes mellitus [Color figure can be viewed at wileyonlinelibrary.com]

To better understand how sympathetic dominance and upregulation of the HPA axes influence carbohydrate metabolism, in this article, we have adapted a version of the integrative model of T2DM proposed by Roden and Shulman^42^ (Figure 3). These shifts in autonomic activity and cortisol release have a widespread impact across the body—affecting immunity, appetite and metabolism. Here, we use a simplified scheme focusing on only the metabolic abnormalities driving insulin resistance upon sleep deprivation. We suggest the classical triumvirate of T2DM (hepatic and muscle insulin resistance and β‐cell dysfunction) can be understood in terms of SNS dominance and elevated cortisol driving lipolysis.^42^ While this model presents a simplified view of how sleep deprivation causes T2DM, we should note the effects of S factor accumulation likely go beyond cortisol and autonomic activity with observed changes in other metabolic hormones such as leptin.^9^ However, it has been difficult to distinguish if these changes are due to circadian abnormalities or S factor accumulation. Further characterization of the effects of somnogens and oxidative stress on these different hormonal axes may enrich our model in the future and are subject to current study.

### 1.3 Role of circadian misalignment in T2DM

The other arm of our 2‐pronged model shown in Figure 1 suggests circadian misalignment plays an important role in driving the pathogenesis of T2DM. The circadian rhythm is driven by a transcription–translation feedback loop involving 4 core clock genes—CLOCK, BMAL1, PER and CRY—oscillating cyclically with a period of about 24 h.^44^ This endogenous rhythm is produced by the master clock in the hypothalamic suprachiasmatic nucleus (SCN), but in addition all cells in peripheral organs also have a cellular, intrinsic circadian clock.^11^ The SCN, entrained primarily by light via the retinohypothalamic tract, uses neuroendocrine pathways to synchronize clocks in other brains areas as well as peripheral organs.^44^ Thus, all clocks throughout the body are synchronized to the SCN and to light‐dark cycles of the environment. These peripheral clocks influence local tissue function and hence, based on the light‐dark cycle, our body anticipates and prepares for various behaviours. These anticipations are critical in metabolism with glucose tolerance and insulin sensitivity peaking in the morning.^45^ This means disruptions to our circadian rhythm can affect insulin sensitivity, increasing the risk of T2DM. In line with this, large‐scale GWAS studies have shown polymorphisms in many clock genes such as CLOCK, BMAL1 and CRY increase the risk of T2DM.^8^ Furthermore, experiments using the forced desynchrony protocol, in which sleep duration is not affected but participant's behavioural cycles lie outside the range of entrainment, show circadian misalignment alone can drive insulin resistance, independent of sleep deprivation.^46^

**Figure 4:**
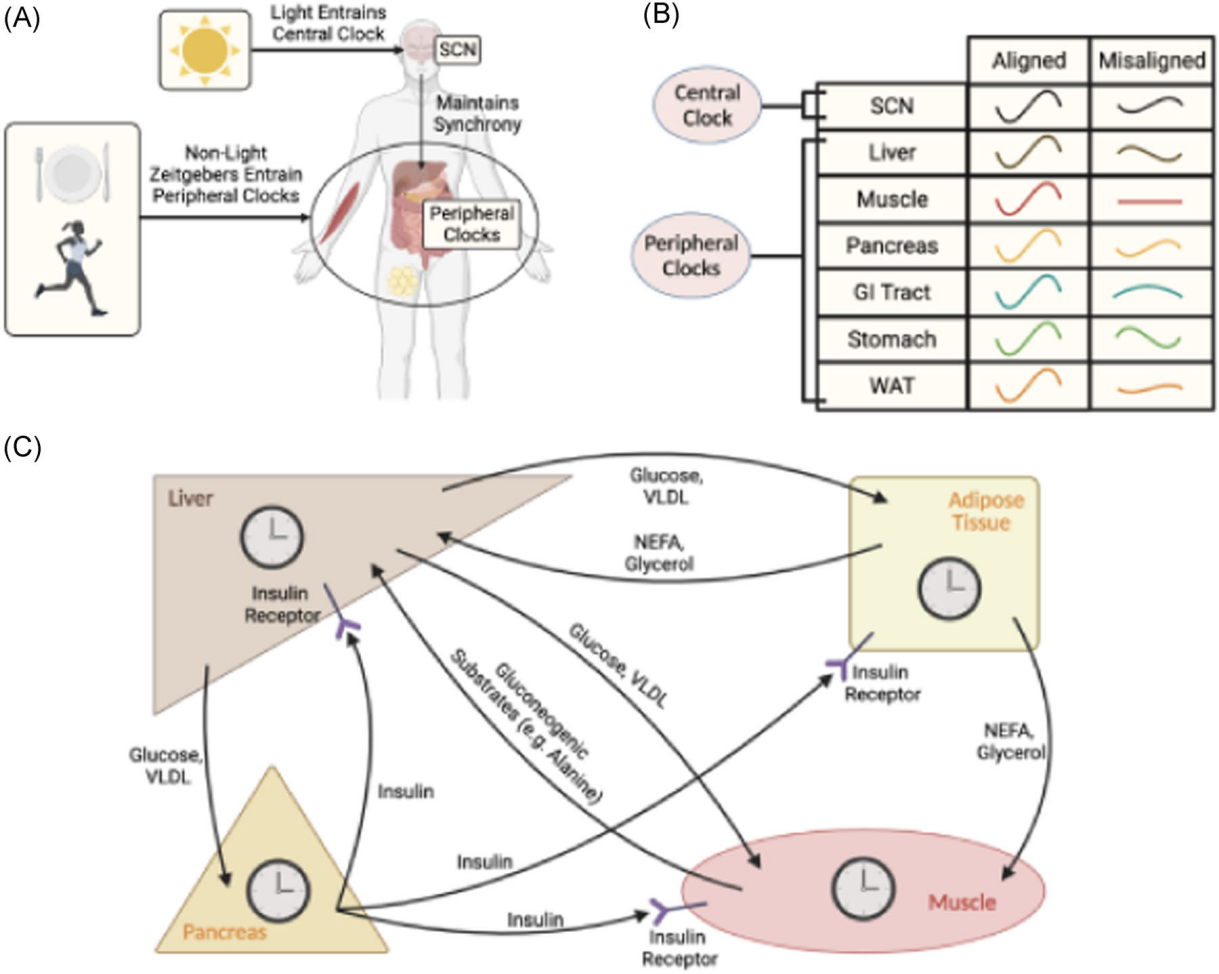
Circadian misalignment and its impact on carbohydrate metabolism. (A) Light and other zeitgebers must be aligned to maintain synchrony between central and peripheral clocks. (B) If synchrony is maintained, the circadian rhythm of the central SCN and peripheral clocks are in‐phase (see aligned condition). If our behavioural cycles do not align with external light cycles, then the amplitude of SCN circadian oscillations is lower, impairing SCN‐mediated anticipatory metabolic processes (e.g., cortisol, growth hormone release). This reduction in central rhythm amplitude as well as the mismatch in behavioural and external light cycles result in desynchrony between not only the SCN and peripheral clocks but also between the peripheral clocks (see misaligned condition). (C) Key metabolic organs are undergoing constant fluxes in metabolites, with different processes being favoured at different times (note: not all the fluxes are shown). This means misalignment across the activity of the organs, due to peripheral desynchrony between the circadian clocks, can impair metabolic function resulting in ectopic lipid accumulation as well as elevated levels of plasma glucose and TAG. Over longer periods of time this can lead to glucolipotoxicity, impairing β‐cell function. GI, gastrointestinal tract; SCN, suprachiasmatic nucleus; VLDL, very low‐density lipoprotein [Color figure can be viewed at wileyonlinelibrary.com]

These circadian effects on glucose tolerance are primarily driven by peripheral clocks, kept in synchrony by the SCN, mediating anticipatory changes that pre‐empt our behavioural cycles. A summary of the metabolic effects of different circadian genes in peripheral tissues is shown in Table 2. We should also note some of the neuroendocrine mechanisms used by the SCN to maintain peripheral clock synchrony may directly influence metabolic processes.^11^ For example, the SCN directly signals to autonomic nerves as well as driving cortisol, growth hormone and melatonin release—all of which separately affect insulin sensitivity.^11^ These anticipatory rhythms are synchronized to light cycles, but the emergence of artificial light has meant our behavioural cycles no longer need to adhere to sunlight. Therefore, as we stay awake for longer, we subject our body to various activities it is not prepared for, the most studied of which is food intake (Figure 4). Supporting this theory, Satchin Panda's group used an app to study the timing of food intake in healthy adults.^47^ They showed most individuals consume food throughout the day, with a mean eating duration of almost 15 h, consuming over 75% of their calories after midday. This constant exposure to nutrients, especially later in the day when glucose tolerance is lower, reduces our ability to efficiently metabolize food. This could subsequently lead to elevated levels of plasma glucose and triglycerides, a common occurrence in shift workers, resulting in ectopic lipid accumulation as well as glucolipotoxicity which can both contribute to the onset of T2DM (Figure 3).^48^, ^49^ In line with this theory, reducing the eating duration to earlier in the day has been shown to improve glucose tolerance in pre‐diabetic individuals, even without weight loss.^50^ Aside from this inability to anticipate activity, misalignment of our SCN rhythm and behavioural cycles may lead to desynchrony between the peripheral clocks locally regulating energy metabolism and the central clock regulating behaviour and eating, contributing to T2DM.^21^ As food, exercise and temperature have all been shown to be zeitgebers of various peripheral clocks, when behavioural cycles do not match patterns of light, the non‐photic zeitgebers and SCN ‘pull’ the peripheral clocks in different directions.^8^, ^21^ As the different peripheral tissues are entrained by different inputs to varying extent, circadian misalignment may result in desynchrony of the peripheral clocks. This problem is compounded by the observation that feeding entrains the so‐called central food‐entertainable clock (FEC). The FEC then drives anticipatory behaviours to food such as releasing cortisol, affecting rhythms of peripheral clocks, and further worsening the clock deschrony.^51^, ^52^ However, further investigation into the FEC have proven extremely difficult as the anatomical site and genes controlling this central oscillator remain unclear.^53^

**Table 2:** Summary of some of the metabolic effects of different clock genes in peripheral tissues based on ablation studies

Model	Tissue of ablation	Ablated gene	Tissue of interest	Finding	References
Mice	Pancreas (Pdx1‐Cre)	Clock	Pancreas	Ablation reduces insulin secretion and impairs glucose tolerance	Marcheva et al.^54^
Mice	Pancreas (Pdx1‐Cre)	Bmal1	Pancreas	Ablation reduces insulin secretion and impairs glucose tolerance	Marcheva et al.^54^
Mouse Fibroblast Cells	Global	Cry1/2	Liver	Ablation increases glucocorticoids signalling, increasing gluconeogenesis	Lamia et al.^55^
Mice	Global	Cry1	Liver	Ablation reduces FOXO1 degradation, increasing gluconeogenesis	Jang et al.^56^
Mouse Myoblast Cell Line	Muscle Cells in Culture	Clock	Muscle	Knockdown reduces SIRT1, reducing insulin signalling pathway	Liu et al.^57^
Mouse Myoblast Cell Line	Muscle cells in culture	Bmal1	Muscle	Knockdown reduces SIRT1, reducing insulin signalling pathway	Liu et al.^57^
Mice	Muscle (Mlc1f‐Cre)	Bmal1	Muscle	Knockout reduces GLUT4 translocation reducing glucose uptake, pyruvate dehydrogenase was also reduced	Dyar et al.^58^
Mice	Liver (Albumin‐Cre)	Bmal1	Liver	Rhythmic expression of GLUT2 was lost, causing hypoglycaemia during fasting	Lamia et al.^59^
Mice	Global	Clock	Adipose	The levels of ATGL and HSL were reduced, favouring adiposity	Shostak et al.^60^
Mice	Global	Bmal1	Adipose	The levels of ATGL and HSL were reduced, favouring adiposity	Shostak et al.^60^

Recently, Andreas Kalsbeek's group provided evidence for this peripheral desynchrony theory by using a shift‐work animal model in which rats were only fed at circadian night in light. They showed, when given a high‐fat diet chronically these animals developed glucose intolerance^61^ and hepatic steatosis,^62^ unlike controls. When these light‐fed rats were then switched back to ad libitum feeding and biopsies were performed at different tissues, specific clock genes had only recovered in some tissues but not others.^63^ One potential issue with this study however is the use of the time‐restricted feeding protocol (TRF) which may artificially increase the ability of food to act as zeitgeber by imposing non‐physiological, longer fasting periods. In line with this, distributing food intake across the restricted feeding period to resemble normal behaviour more closely had a much smaller effect on phase shifts observed in PER2.^64^ Figure 4 shows how circadian misalignment with behavioural cycles can impair anticipatory metabolic processes, hence contributing to T2DM. Aside from the timing of food intake, peripheral desynchrony may also arise due to other changes in behaviour. For example, rats whose wheel‐running activity was restricted to the sleep period showed, even after 4 weeks of recovery, only some clock genes in the liver had returned to normal.^65^ While this study did not probe gene expression in other peripheral sites, future studies may confirm the desynchrony.

### 1.4 Future insights

While the scheme we proposed (Figure 1) is useful in helping us untangle the effects of SCRD on the intertwined circadian and sleep processes, we are only beginning to understand its role in T2DM. Though the relative sympathetic dominance after adenosine and ROS build‐up is clear, whether the increases in these factors after sleep deprivation is sufficient to drive T2DM remains unresolved. In addition to this, though the autonomic nervous system and cortisol can influence metabolic processes, direct evidence implicating these processes with T2DM is limited. That said, epidemiological studies show those with Cushing's disease^66^ or hypertension^67^ (associated with SNS upregulation^33^) have a higher risk of T2DM. Though this epidemiological data only serves to establish a correlation, more direct investigation into the effects of the autonomic nervous system and cortisol on T2DM may support our theory. On the circadian arm of our model, the main difficulty has been investigating metabolic effects of peripheral clocks in the absence of specific Cre‐lines.^68^ Generally, some Cre expression in nontarget sites would not be a major problem but the widespread expression of clock genes means virtually every cell expressing the recombinase would be affected.^68^ In the future, the use of transgenic viral delivery systems carrying tissue‐specific promoters promises to increase the spatiotemporal resolution.^68^ New optogenetic approaches using photo‐sensitive clock proteins, such as Arabidopsis cryptochrome 2, may allow us to use light to directly affect clock function with a high, reversible temporal precision.^68^ These new approaches may allow us to investigate the consequences of specific peripheral clock manipulations on metabolism.

Aside from investigating the two arms separately, we should also attempt to characterize the interactions between both branches in greater detail. It is clear the S and C factors interact with each, with dysfunction in either arm causing abnormalities in the other.^24^ For example, sleep deprivation alone may upregulate the HPA axis causing the release of cortisol which can affect the circadian rhythm which then influences diurnal release of cortisol to alter ROS and hence the S factor. In line with this idea of a highly interconnected S and C process, recent studies show clock genes have redox properties^69^ and may be directly affected by adenosine accumulation,^25^ providing a viable molecular mechanism of S–C interaction. Exploring these complex S–C interactions will likely yield critical insights to better understand T2DM.

## 2 CONCLUSION

The association between T2DM and SCRD is clear but, despite the substantial mechanistic insight discussed above, few clinical guidelines take these principles into account when diagnosing or treating diabetes. Recent evidence indicates simply changing the timing of feeding or exercise improves T2DM, highlighting the importance translating this study into medical practice.^50^ Though our model only represents a starting point to understand SCRD contribution in T2DM, future investigations may elucidate the finer details underlying the circadian misalignment, S factor accumulation as well as the interaction of the two axes. By doing this, we could pioneer new drugs that directly modulate the circadian clock or S factor to reduce the impact of SCRD in driving T2DM. The massive commercial and military importance of ‘conquering’ sleep has meant, even now, drugs directly targeting clock genes are being developed with some of them reducing obesity and hyperglycaemia.^70^ Further research into the metabolic implications of sleep and circadian rhythms may represent an important avenue in preventing, diagnosing, and better managing T2DM.
